# Maternal obesity programmes offspring development of non-alcoholic fatty pancreas disease

**DOI:** 10.1016/j.bbrc.2010.02.057

**Published:** 2010-03-26

**Authors:** Jude A. Oben, Trusha Patel, Angelina Mouralidarane, Ann Maj Samuelsson, Phillippa Matthews, Joaquim Pombo, Maelle Morgan, Chad Mckee, Junpei Soeda, Marco Novelli, Lucilla Poston, Paul Taylor

**Affiliations:** a Centre for Hepatology, University College London, Royal Free Hospital, London NW3 2PF, UK; b Department of Gastroenterology, Guy’s and St. Thomas’ Hospital, London, UK; c King’s College London, Division of Reproduction and Endocrinology, London, UK; d Department of Pathology, University College London, London, UK

**Keywords:** Maternal obesity, Developmental programming, Pathogenesis, Non-alcoholic fatty pancreas

## Abstract

**Background and aims:**

The prevalence of pancreatic adenocarcinoma (PAC) parallels rising rates of obesity and dysmetabolism, a possible link being non-alcoholic fatty pancreas disease (NAFPD). We have recently shown that maternal obesity programmes the development of a dysmetabolic and fatty liver (non-alcoholic fatty liver disease, NAFLD) phenotype in adult offspring. Since the pancreas and liver originate from the same embryonic bud, it is plausible that maternal obesity may similarly programme the development of NAFPD. Our objective was to determine the effect of maternal obesity on development of NAFPD in offspring and ascertain contributions of the intra/extra-uterine periods.

**Methods:**

Female C57BL/6J mice were fed either a standard chow (3% fat, 7% sugar) or a hypercalorific diet (16% fat, 33% sugar) for six weeks prior to mating and throughout pregnancy and lactation. Female offspring were cross-fostered for suckling to dams on the same or opposite diet to yield four groups: offspring of lean suckled by lean dams (*n* = 6), offspring of obese suckled by obese dams (*n* = 6), offspring of lean suckled by obese dams (*n* = 5) and offspring of obese suckled by lean dams (*n* = 6). All offspring were weaned onto a standard chow diet at 21 days and sacrificed at 3 months post-partum for tissue collection.

**Results:**

Offspring subjected to an adverse suckling environment showed significant increases in body weight, pancreatic triglyceride content, TGF-β, collagen gene expression and SBP at rest along with an enhanced restraint stress response, indicating a dysmetabolic and NAFPD phenotype.

**Conclusions:**

Developmental programming is involved in the pathogenesis of NAFPD and appears to be largely dependent on an adverse extra-uterine environment.

## Introduction

The prevalence of pancreatic cancer in affluent countries is rising and may be due to rising rates of obesity [1]. In the USA the incidence of pancreatic (body/tail) cancers increased by 46% between 1973 and 2002, in parallel with rising rates of obesity [2,3]. Emerging evidence suggests that maternal obesity may programme offspring obesity and dysmetabolism [4] such that the phenomenal rises in the rates of obesity within the last few decades may not just be due to the ready availability of cheap energy dense foods but may also be due to transgenerational amplification of obesity through epigenetic mechanisms [5–7]. This is of importance because obesity amongst women of reproductive age is rising as with the general population, with some 29% of women, aged 20–39 years, in the USA reported to be obese [8]. Additionally, some series have reported rates of obesity of 20% in pregnant women at the time of registration antenatal care [9]. A recent report indicates that the incidence of maternal obesity in first trimester of pregnancy has more than doubled in less than 20 years [10].

The link between obesity and pancreatic cancer may be obesity-induced non-alcoholic fatty pancreas disease (NAFPD). This relatively recently described disease entity [11,12], describes a pancreatic phenotype ranging from deposition of fat in the pancreas through fat deposition with inflammation, resultant pancreatic fibrosis and the possibility of pancreatic cancer. This pancreatic phenotype is similar to that of obesity-induced liver disease, non-alcoholic fatty liver disease (NAFLD) which describes a spectrum from hepatic steatosis through steatohepatitis to cirrhosis, and primary hepatocellular cancer [13–15]. We have recently shown that maternal obesity programmes the development of a dysmetabolic and fatty liver phenotype in adult offspring [16]. As the pancreas and liver originate from the same embryonic bud, it is plausible that maternal obesity may similarly programme the development of NAFPD.

Using a similar protocol to that previously published [16], our objective in this study therefore, was primarily to determine whether maternal obesity programmes the development of NAFPD in adult offspring. Additionally, we assayed the relative contributions of an adverse extra-uterine compared to an adverse intra-uterine environment in the development of this phenotype by cross-fostering a subset of offspring to dams on the opposite diet during the suckling period. Metabolic dysfunction was then determined by analysis of physiological, biochemical and histological data, along with tissue triglyceride content. Markers of pancreatic injury and fibrosis were also assayed.

## Methods

*Animal husbandry.* Proven breeding female C57BL/6J (Charles River Laboratories, UK), following acclimatization, were randomly allocated to either standard chow (7% simple sugars, 3% fat, 50% polysaccharide, 15% protein [w/w] RM1, Special Dietary Services, energy 3.5 kcal/g) or an obesogenic diet (10% simple sugars, 20% animal lard, 28% polysaccharide, 23% protein [w/w], Special Dietary Services, energy 4.5 kcal/g). The obesogenic diet was rendered more palatable through supplementation with Nestle sweetened condensed milk (approx 55% simple sugar, 8% fat, 8% protein [w/w]) mixed with mineral mix (SDS Batch 31801, 125 mg/pot) and fed *ad libitum.* Body weights and food intake were recorded weekly. All animals were treated in accordance with The Animals (Scientific Procedures) Act 1986 guidelines. Following 6 weeks on the allocated diet, females were mated with C57BL/6J males from the same litter. Conception was determined by the formation of a vaginal plug and pregnant dams were maintained on their respective diets throughout gestation and lactation. During pregnancy, maternal weight and dietary intake were recorded weekly. Eight hours after delivery litters were standardized to six pups with an equal number of males and females wherever possible, to ensure adequate milk supply. Dams along with litter sizes less than 4 were also sacrificed.

*Cross-fostering experiments.* Following standardization of the pups as above, female offspring were cross-fostered for suckling to a dam on either the same diet as their biological mother or a dam on the opposite diet, to produce four offspring groups; group 1 – offspring of lean suckled by lean dams (*n* = 6); group 2 – offspring of obese suckled by obese dams (*n* = 6); group 3 – offspring of lean suckled by obese dams (*n* = 5) and group 4 – offspring of obese suckled by lean dams (*n* = 6). The lean and obese dams as depicted in groups 1 and 2 did not suckle their own offspring to control for the cross-fostering stress possibly imposed on groups 3 and 4 as above.

At 21 days post-partum, offspring from all groups were weaned onto standard chow. Body weights and food intake were measured weekly. Offspring had access to food and water *ad libitum* and were maintained in a 12-h light/dark cycle, in a thermostatically controlled environment (25 °C) throughout.

*Total body weight and pancreas weight.* At 3 months of age offspring were weighed, humanely sacrificed, pancreata were removed weighed and snap frozen in liquid nitrogen.

*Radiotelemetry.* Blood pressure was measured using carotid artery placement of radiotelemetric probes (TA11PA-C10, O.D 0.4 mm, Data Science International Inc., St. Paul, MN) under general anaesthetic at 3 months of age (ketamine and medetomidine). Following at least 1 week post-surgery recovery, blood pressure, heart rate and activity were measured using the Dataquest IV telemetry system, in freely moving offspring over a 24-h period.

*Stress data.* At 3 months of age, telemetered offspring were also exposed to 20 min restraint stress after which they were returned to their home cage for a 120 min recovery period. Heart rate and blood pressure measurements were assayed every minute over the 20 min stress period and then every 5 min over a 120 min recovery period.

*Expression of pancreatic injury and fibrogenesis markers.* Following sacrifice at 3 months of age, the pancreas was dissected out, weighed and snap frozen in liquid nitrogen. The tissue samples were stored at −80 °C. Pancreas tissue was analyzed for markers of fibrosis, namely – collagen type 1-α2 and TGF-β. Quantitative real time PCR was performed to determine gene expression as previously described [16]. Primer sequences were as in Table 1.

*Tissue triglyceride analysis.* An adaptation of the Folch Method and triglyceride assay reagents (Roche Diagnostics) was used to determine murine whole pancreas tissue triglyceride content.

*Statistical analysis.* Statistical analysis was conducted using GraphPad Prism 5. Data are expressed as mean ± SEM. Significance was determined using unpaired *t*-test or one-way ANOVA as appropriate and significance accepted at *p* < 0.05.

## Results

### Maternal obesity during pregnancy or suckling promotes offspring obesity

**Fig. 1:**
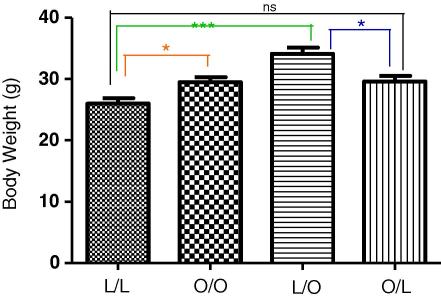
Body weight at 3 months post-partum. Offspring of lean suckled by lean (L/L); offspring of obese suckled by obese (O/O); offspring of lean suckled by obese (L/O) and offspring of obese suckled by lean (O/L). Values are expressed as mean ± SEM, *n* = 4–6/group; *p < 0.05, ***p < 0.0001, ns – not significant.

At 3 months post-partum, offspring of obese suckled by obese dams had significantly greater body weights compared to offspring of lean suckled by lean dams (Fig. 1, *p* < 0.05). In addition, offspring of lean suckled by obese dams had significantly increased body weights compared to offspring of lean suckled by lean dams (Fig. 1, *p* < 0.0001). There was importantly, no statistical difference in body weight between offspring of lean suckled by lean dams and offspring of obese suckled by lean dams. Therefore, maternal over-nutrition during pregnancy and the suckling period induces offspring obesity, with as we have shown previously, offspring nutrition in the immediate post-partum period being the more critical determinant of offspring obesity [4,16,17].

### Maternal obesity during pregnancy or suckling promotes offspring pancreas tissue triglyceride content

In parallel with increases in body weight as above, pancreas tissue triglyceride content was significantly increased in offspring of obese suckled by obese dams compared to offspring of lean suckled by lean (Fig. 2A, *p* < 0.0001) and in offspring of lean suckled by obese (Fig. 2B, *p* = 0.0117).

### Pancreatic expression of transforming growth factor-β (TGF-β1) and collagen type 1-α2 as markers of activation of pancreatic fibrogenesis

Compared with offspring of lean suckled by lean dams, offspring of obese suckled by obese dams, had significantly increased relative TGF-β gene expression (Fig. 3A, *p* = 0.0025) as did offspring of lean suckled by obese dams (*p* = 0.0025). Similarly, compared with offspring of lean suckled by lean dams, offspring of obese suckled by obese dams had an elevated but non-significant expression of collagen gene expression whilst lean offspring suckled by obese dams showed a significant increase in collagen gene expression (Fig. 3B, *p* = 0.0052). Importantly, offspring of obese suckled by lean had levels of TGF-β and collagen gene expression no different to control levels in control offspring (offspring of lean suckled by lean). Therefore, offspring exposure to an obesogenic environment immediately post-partum programmes the development of a fatty pancreas with induced fibrogenesis.

### Mechanisms of pancreatic injury

#### Night-time systolic blood pressure as evidence of sympathetic nervous system activation

Since hepatic fibrogenesis involves sympathetic nervous system activation (SNS) [18], it is possible that pancreatic fibrosis similarly involves SNS activation. Given that SNS activation is reflected by nocturnal hypertension [4,16] we therefore sought to determine if maternal obesity influenced offspring night-time systolic blood pressure (NSBP). Compared with offspring of lean suckled by lean, offspring of obese suckled by obese dams had significantly elevated NSBP (Fig. 4A, *p* < 0.0001). Additionally, offspring exposed to maternal over-nutrition during the suckling period only (offspring of lean suckled by obese dams), also had a statistically significant increase in NSBP compared with offspring of lean suckled by lean (Fig. 4A, *p* < 0.0001).

#### Systolic blood pressure response to stress as evidence of sympathetic nervous system activation

SNS activation was confirmed by increases in systolic blood pressure (SBP) of offspring subjected to stress. In offspring of obese suckled by obese, compared with offspring of lean suckled by lean, there was a statistically significant increase in SBP in response to restraint stress (Fig. 4B, *p* < 0.0001). Moreover, offspring of lean suckled by obese dams also had a significantly increased SBP, in response to restraint stress, compared with offspring of lean suckled by lean dams or offspring of obese suckled by lean (Fig. 4B, *p* < 0.0001). Importantly, there was no difference in SBP between offspring of lean suckled by lean and offspring of obese suckled by lean, confirming that an adverse extra-uterine environment is the principal determinant of the hypertensive phenotype induced by maternal obesity.

## Discussion

The results here confirm as previously shown [4,16,17,19] that maternal diet-induced obesity in rodents, most probably through effects on the early post-natal period, induces an obese, hypertensive phenotype in offspring. In these dysmetabolic offspring we observed significant increases in body weight, pancreas tissue triglyceride content, increased pancreatic expression of the fibrogenic markers TGF-β1 and collagen gene along with increases in NSBP and SBP response to restraint. Therefore, we have demonstrated here a hitherto undescribed fatty pancreas phenotype found in association with the programmed obese and hypertensive phenotype. The importance of these findings is that they imply that a pancreas phenotype similar to NAFLD may also be programmed by maternal obesity. Given that NAFLD may lead to hepatocellular carcinoma [14,15], it is conceivable that the rising rates of pancreatic adenocarcinoma may, by extrapolation from NAFLD, be secondary to NAFPD. That NAFPD [11], or at least components of it, like NAFLD appears to be programmable by maternal obesity implies that the rising rates of pancreatic cancer may be due to rising rates of maternal obesity.

In the present study, the expression of TGF-β1 as a marker of pancreatic stellate cell (PSC) activation was also upregulated. There was a significant increase in TGF-β1 expression in the two groups subjected to an adverse extra-uterine environment, compared to the lean control group. A similar trend was observed for collagen expression, suggesting TGF-β1 as the driver for the upregulation of collagen gene expression in our model as is the case for hepatic stellate cells (HSC) [18]. Collagen is the major extracellular matrix component produced on activation of PSC in response to injury and we have shown here an increased expression of collagen type 1-α2 mRNA in offspring exposed to over-nutrition during the suckling period, compared with the lean control group. This implies that as with liver, pancreatic injury may lead to pancreatic fibrosis and perhaps through this to pancreatic cancer. It is though conceivable that as with hepatocellular cancer, pancreatic cancer may arise in the absence of significant fibrosis and that alone, over-exuberant deposition of fat in the pancreas may be sufficient to induce pancreatic cancer [14]. Importantly, pancreatic steatosis has been shown to promote dissemination of pancreatic cancer [20].

A potential mechanism for induction of pancreatic fibrosis may be upregulation of the SNS. SNS activation is implied here by the demonstrated increase in NSBP and by increased SBP in response to restraint stress. NSBP and SBP with stress were significantly increased in offspring subjected to an adverse extra-uterine environment compared with the lean control group. There is evidence that SNS signalling components, such as SNS neurotransmitters, may be pro-fibrotic in NAFLD through their effects on adrenoreceptors expressing hepatic stellate cells [18,21,22]. Whilst this has not been shown for pancreatic stellate cells, it is tempting to speculate a similar pathway exists for PSC and pancreatic fibrosis. Most recently, in a parallel model we have described evidence for the sympathetic origins of hypertension in the juvenile offspring of obese rats, in which persistent sympatho-excitatory hyper-responsiveness is acquired in the early stages of development [19]. A role for adrenergic receptors in the activation of intracellular signalling pathways that lead to mitogenic responses has been described [18,23,24], whilst abnormalities of sympathetic effects, including disturbances of leptin and β3-adrenergic receptor signalling may contribute to the development of obesity and type 2 diabetes in rodents [25]. We hypothesize that the exaggerated leptin surge [17] and increased sympathetic drive [19] we observe in offspring of obese rodents may also contribute to altered pancreatic development post-natally and promote NAFPD in this model [26]. Importantly, increased sympathetic drive has also implicated in proliferation of pancreatic cancer cells [27,28].

In conclusion, these novel findings show that maternal obesity leads to NAFPD and a dysmetabolic phenotype in offspring. Moreover, the suckling period appears to be an important determinant of this phenotype. NAFPD may be the pancreas’ manifestation of the metabolic syndrome and like NAFLD is programmable. By extrapolation from NAFLD, our findings provide a possible explanation for the parallel increase in obesity and pancreatic cancer through NAFPD. The role of leptin and the sympathetic nervous system in the initiation of the NAFPD phenotype during the critical post-natal period will be the focus of future studies.

## Disclosures

None.

## Figures and Tables

**Fig. 2A:**
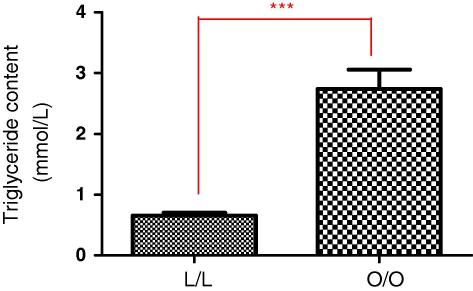
Pancreas tissue triglyceride content at 3 months post-partum. Offspring of lean suckled by lean (L/L); offspring of obese suckled by obese (O/O). Values are expressed as mean ± SEM, *n* = 3–5/group; ***p < 0.0001.

**Fig. 2B:**
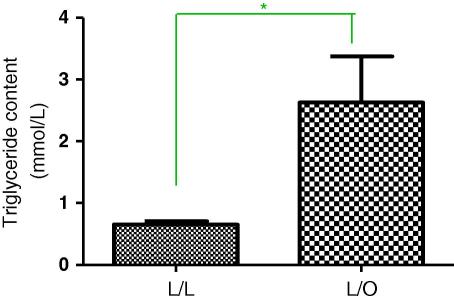
Pancreas tissue triglyceride content at 3 months post-partum. Offspring of lean suckled by lean (L/L); offspring of lean suckled by obese (L/O). Values are expressed as mean ± SEM, *n* = 3–5/group; *p < 0.01.

**Fig. 3A:**
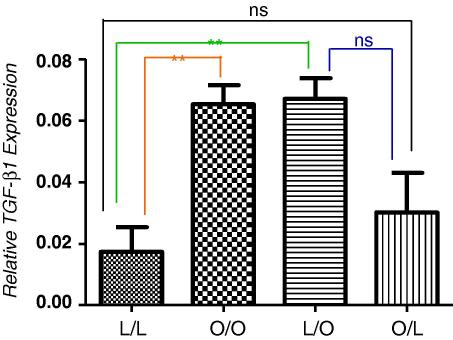
Relative expression of TGF-β mRNA in the pancreas at 3 months post-partum, expressed as a percentage of GAPDH. Offspring of lean suckled by lean (L/L); offspring of obese suckled by obese (O/O); offspring of lean suckled by obese (L/O) and offspring of obese suckled by lean (O/L). Values are expressed as mean ± SEM, *n* = 3–5/group; **p < 0.0025, ns – not significant.

**Fig. 3B:**
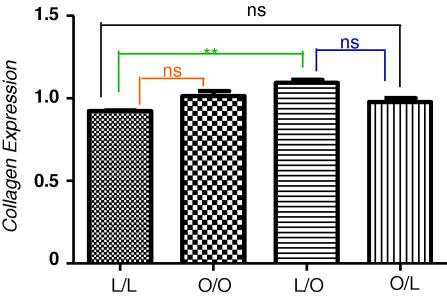
Relative expression of collagen type 1-α2 mRNA in the pancreas at 3 months post-partum, expressed as a percentage of GAPDH. Offspring of lean suckled by lean (L/L); offspring of obese suckled by obese (O/O); offspring of lean suckled by obese (L/O) and offspring of obese suckled by lean (O/L). Values are expressed as mean ± SEM, *n* = 3–6/group; ***p < 0.0001, **p < 0.005; ns – not statistically significant.

**Fig. 4A:**
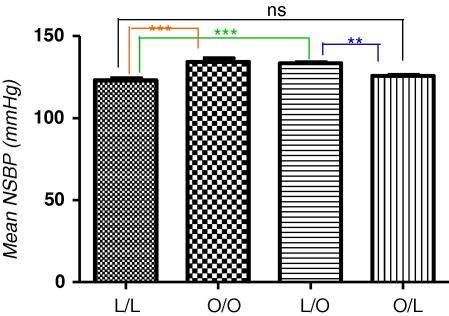
Night time systolic blood pressure at 3 months post-partum. Offspring of lean suckled by lean (L/L); offspring of obese suckled by obese (O/O); offspring of lean suckled by obese (L/O) and offspring of obese suckled by lean (O/L). Values are expressed as mean ± SEM, *n* = 6/group; ***p < 0.0001, **p < 0.001; ns – not statistically significant.

**Fig. 4B:**
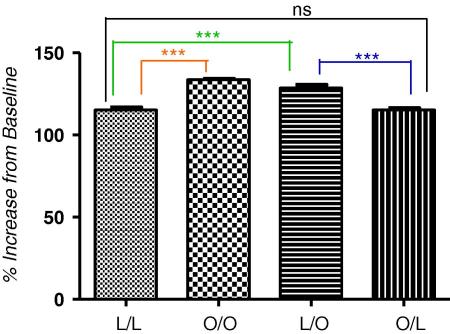
Systolic blood pressure as percentage of baseline in offspring subjected to a 20 min restraint stress at 3 months post-partum baseline is denoted as 100%. Offspring of lean suckled by lean (L/L); offspring of obese suckled by obese (O/O); offspring of lean suckled by obese (L/O) and offspring of obese suckled by lean (O/L). Values are expressed as mean ± SEM, *n* = 4–5/group; ***p < 0.0001; ns – not statistically significant.

**Table 1:** Primer sequences, expected weights and annealing temperatures.

Collagen type 1α	Sense primer: 5′-GAACGGTCCACGATTGCATG-3′ Antisense primer: 5′-GGCATGTTGCTAGGCACGAAG-3′ Annealing temp: 55 °C	Expected weight: 167 bp
TGF-β1	Sense primer: 5′-AAAATCAAGTGTGGAGCAAC-3′ Antisense primer: 5′-CCACGTGGAGTTTGTTATCT-3′ Annealing temp: 59 °C	Expected weight: 224
GAPDH	Sense primer: 5′-CACAATTTCCATCCCAGACC-3′ Antisense primer: 5′-GGGTGCAGCGAACTTTATTG-3′ Annealing temp: 60 °C	Expected weight: 93 bp
